# 3D bioprinting of cardiac tissue: current challenges and perspectives

**DOI:** 10.1007/s10856-021-06520-y

**Published:** 2021-05-06

**Authors:** Brian Kato, Gary Wisser, Devendra K. Agrawal, Tim Wood, Finosh G. Thankam

**Affiliations:** Department of Translational Research, Western University of Health Sciences, Pomona, CA USA

## Abstract

Demand for donor hearts has increased globally due to cardiovascular diseases. Recently, three-dimensional (3D) bioprinting technology has been aimed at creating clinically viable cardiac constructs for the management of myocardial infarction (MI) and associated complications. Advances in 3D bioprinting show promise in aiding cardiac tissue repair following injury/infarction and offer an alternative to organ transplantation. This article summarizes the basic principles of 3D bioprinting and recent attempts at reconstructing functional adult native cardiac tissue with a focus on current challenges and prospective strategies.

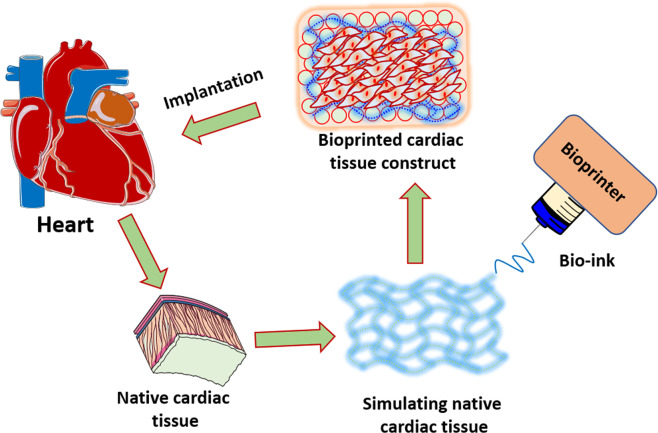

## Introduction

Mortality due to cardiovascular disease (CVD) has been on a global incline due, in large part, to a shortage of donor organ supply. Heart transplantation is often needed to treat CVD because the heart tissue is unable to regenerate cardiomyocytes. Specifically, a lack of blood flow to the myocardium (myocardial infarction) results in scarring and permanent loss of cardiomyocytes which can ultimately lead to heart failure and/or arrythmias [1]. Currently, heart transplantation remains the most effective option for end-stage cardiovascular diseases [2]. Though a number of subjects are fortunate to receive a donor heart, many patients die while awaiting a donor thus making organ shortage a major issue in prolonging the lives of patients with advanced CVD. Furthermore, new heart transplant listings increased by 57% between 2005 and 2016 while the number of patients waiting to undergo heart transplantation increased by 127% over the same period; showing an increasing demand for donor hearts [3]. Many hurdles exist in achieving successful heart transplantation including procurement of the donor heart and post-transplant organ rejection. While technologies such as ex-vivo heart perfusion have made it possible to deliver hearts to geographically distant recipients, treating organ rejection in a sustainable way (e.g. – without immunosuppression) remains challenging [4]. An emerging approach for addressing the growing demand for heart transplantation is 3D bioprinting of cardiac tissue due to its potential to repair cardiac tissue. In addition to restoring the function of infarcted tissue, 3D bio-printed cardiac tissue that is derived from autologous cells is less likely to trigger an immune response. Given these possible benefits, 3D bioprinting has the potential to develop cardiac tissue constructs for the treatment of CVD.

Recent approaches to three-dimensional cardiac tissue construction have yielded promising results, indicating its potential for creating alternatives to heart transplantation. These methods utilize biomaterials such as alginate, gelatin, collagen, and decellularized extracellular matrix (ECM) in conjunction with bioprinting methods such as extrusion, inkjet, laser-assisted, stereolithography, or scaffold-free [5]. Depending on the type of cardiac tissue (e.g. – myocardium, valves, blood vessels, connective tissue) being created, specific combinations of biomaterials and bioprinters are selected. For example, in order to create an environment conducive to cardiac cell proliferation and crosslinking, two criteria for creating functional cardiac tissue, Lee et al. utilized extrusion-based bioprinting along with collagen/ECM and alginate resulting in a 3D porous structure with minimal cytotoxicity [6]. Successful experiments such as this have been reported with their own unique advantages and limitations in recreating the morphology, mechanical parameters, protein expression, and electrophysiological characteristics of adult native cardiac ECM. Since in vitro tissue closely resembling the native cardiac environment is likely to recapitulate normal cardiac function in vivo, these four general groups are further divided into subcategories to determine which methods are the most effective. For instance, mechanical properties are split into contractile force and conduction velocity while electrophysiological characteristics are divided into resting membrane potential and upstroke velocity. Herein, the focus is on the technology that yielded cardiac tissue constructs simulating adult native cardiac tissue.

## Mimicking the native structure of heart tissue

The unique interplay between adult native cardiac ECM components and cardiac cells as well as the physical characteristics of adult cardiac tissue are necessary for the proper function of 3D-printed structures in vivo. In human cardiac development, these properties are cultivated in sequence from fetus to adult. For example, the conduction velocity of immature fetal CMs increases from 0.1–0.2 to 0.3–1 m/s as they develop into the mature phenotype. Other features such as contractile force, resting membrane potential, and gene expression change in order to promote normal function. Despite this understanding of cardiac tissue maturation, bioprinting efforts have mostly resulted in phenotypes that resemble fetal tissue or tissue lacking all of the attributes of mature heart tissue (e.g. - mechanical, electrophysiological, or gene expression) [7]. Several studies have shown that engineered tissue is more likely to reach a mature state when parameters such as scaffold stiffness and choice of cell types are optimized.

In order to pump five liters of blood per minute through the body, the average human heart requires not only the structural components to withstand the stress, but also the contractility to deliver the required pressure [8]. For this reason, achieving the proper elasticity, stiffness, and cell density is an important goal in the bioprinting of functional cardiac tissue. For instance, Lee et al. conducted a study using human induced pluripotent stem cell-derived cardiomyocytes (hiPSC-CMs) in crosslinked gelatin hydrogels. The contraction velocity and sarcomere organization of the resulting tissue was compared across three hydrogel stiffness levels – low, intermediate, and high. The results showed that only the intermediate gel (9 kPa), which is within the range of native cardiac tissue stiffness (8–11 kPa), yielded tissue possessing organized sarcomeres with increased contractile force. Similarly, Edalat et al. used embryonic stem cell-derived CMs combined with hydrogels of varying type I collagen concentrations (0, 3, and 6 mg) to construct cardiac tissue. The results showed that the 3 mg hydrogel increased myosin regulatory light chain 2 (MLC2v) which affects sarcomere and CM organization [9]. The fact that these features were only observed in tissue derived from gels within a specific range of stiffness or collagen concentration, indicates that regulation of the physical environment of cells is required to optimize the mechanical properties of cardiac tissue.

Another vital feature of native CMs is automaticity to induce spontaneous rhythmic contractions that reliably circulate blood to the body. Mannhardt et al. reported spontaneously beating cardiac tissue generated from hiPSC-CMs in hydrogels where the contractile force and spontaneous beating were similar to normal adult CMs (i.e. – 40–80 mN/mm^2^) [7]. In contrast, Liu et al. used GelMA and human embryonic stem cell-derived CMs (hESC-CMs) which resulted in a contractile force in the range of immature fetal CMs (i.e. – 0.08–4 mN/mm^2^) [7]. The difference in contractile force generated from hiPSC-CMs versus hESC-CMs suggests that the specific type of cell used to create tissue affects its functionality.

In addition, the expression of various proteins at particular times during the development of cardiac tissue determines its maturation and function. For example, type III collagen is much more abundant than type I collagen in immature fetal heart cells whereas type III collagen and type I collagen exist at a ratio of approximately 2:1 in mature adult heart cells. Type I collagen is fibrous and provides structure while type III collagen is globular and confers elasticity. This shift in the relative amounts of these proteins during maturation emphasizes the importance of appropriately timed gene expression to achieve full cardiac tissue function. The unique phenotypes of type I/III collagen have been attributed to their respective amino acid sequences as well as the aggregation of triple helices within their structures [10]. Both types of collagen originate from fibroblasts which populate the four chambers of the heart during development. Subsequently, they begin secreting the ECM components necessary for CM growth [11, 12]. Type III collagen works in concert with type I collagen to create a flexible structure capable of withstanding repeated pressure fluctuations [13]. However, elastin (another ECM protein) is needed within this blend of proteins to deliver elasticity for expansion and contraction [14]. Within the native ECM environment, specific ECM proteins are needed to provide distinct tissue phenotypes for normal cardiac function.

Another important collagen is type IV, which is a major part of the basement membrane [15]. Collagen IV not only provides physical support but also serves as a tissue compartment barrier and scaffold for cell attachment and migration [16]. Moreover, evidence suggests that type IV collagen is directly related to coronary artery stiffness (an increase in collagen type IV expression leads to decreased arterial compliance) which aligns with its role as a structural protein [17, 18]. Other proteins including fibronectin and laminin play crucial roles in cell adhesion/migration and intracellular signaling [19, 20]. These ECM proteins possess unique characteristics to ensure appropriate gene signaling, protein interactions, and structural balance within cardiac tissue.

**Fig. 1:**
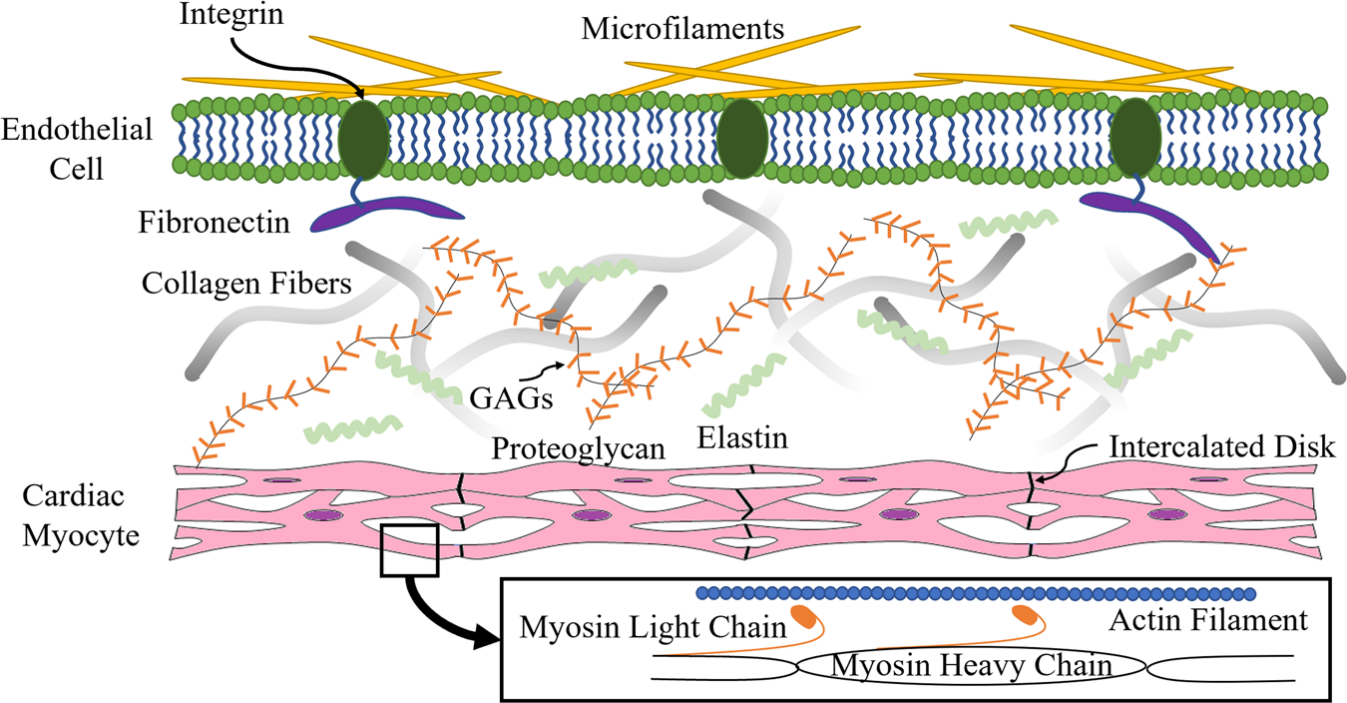
Native Cardiac ECM. Fibronectin (purple) plays a role in cell adhesion by anchoring cells. Integrins (dark green) work to bind cells to the ECM. Microfilaments (yellow) help with cell movement. Elastin (light green) provides elasticity to tissues. Proteoglycans are composed of GAGs (orange) which bind cations and water; and are involved in cell communication and regulation. Collagen fibers (gray) provide elasticity and support to tissues. Myosin light chain (dark orange) binds to actin (blue) whereas myosin heavy chain aids in generating a contractile force

Furthermore, proteins such as connexin-43 (an intercellular junction protein) are integral in fostering gap junction formation for adequate cell-to-cell conduction [21]. Gap junctions connect the cytoplasm of adjacent cells to allow the flow of electrical current and biochemicals that are required to generate normal contractility in the heart. Wang et al. confirmed the presence of connexin-43 in extrusion-bioprinted tissue using CM-laden hydrogel; three weeks following printing, CMs aligned properly and upregulated connexin-43 as well as exhibited a synchronous contractile force of 6.8 mN. This study demonstrated that gene expression status triggers the necessary cellular changes such as cell alignment and gap junction formation to promote optimal cardiac tissue function. The overview of native cardiac tissue including the key ECM components is shown in Fig. 1.

## 3D bioprinting techniques

Bioprinting was first demonstrated in 1988 by loading a bioink solution consisting of collagen and fibronectin into a commercially available Hewlet-Packard inkjet printer [22]. The inkjet method works by loading cells into a cartridge so that the bioprinter deposits organized layers of cells into a scaffold/medium such as a hydrogel. The scaffolds provide attachment sites and nutrients for the cells to proliferate and mature into the desired tissue. Inkjet bioprinting achieves up to 50 μm resolution; however, being a pressure-based system it accommodates very low cell density (less than 10^6^ cells/mL) [5]. Inkjet technology remains limited in its application to complex cell-laden structures despite its low cost, high printing speed and cell viability [23]. The ability to create the types of detailed structures that mimic native tissue is necessary for clinical application; however, it is difficult to achieve with inkjet technology. Recently, methods such as printing with multiple bioinks have enabled the generation of complex tissue structures with inkjet technology. For example, Negro et al. created a microfluidic network with the use of two bioinks: non-digestible and digestible. The digestible bioink was alginate-based which was designed to be catabolized by alginate lyase while the non-digestible bioink remained intact when exposed to the enzyme. Following the layer-by-layer printing, where digestible bioink was used in locations reserved for perfusion and non-digestible bioink was used for the surrounding portion, the structure was exposed to alginate lyase which created microfluidic pathways. In order to deposit cells inside the microfluidic patterns the alginate-based bioink was modified with host cells. After the enzymatic digestion, the cells retained the printed patterns leaving a complex cell-laden structure. Studies such as this, shed light on the possibility of using inkjet technology for complex clinical applications.

Extrusion-based bioprinting releases biomaterials in cylindrical filaments to construct 3D tissues layer-by layer. Similar to inkjet technology, it utilizes pressure to expel biomaterials in a precise orientation. Although extrusion-based techniques are capable of using high cell density biomaterials, the moderately high cost, lower resolution compared to inkjet, slow printing speed, and 40–80% cell viability after printing offers challenges [24]. However, it has shown promise in printing thick myocardial constructs, heart valves, and blood vessels [5]. Additionally, biocompatible materials such as alginate have been employed to create complex structures [25].

Laser-assisted bioprinting techniques such as laser-induced forward transfer (LIFT) employ two parallel slides, between which, laser-absorbing metal covered with biomaterial is placed. As the metal absorbs the laser pulses, it evaporates, causing the biomaterial to fall onto the lower slide in a specific position. Despite lower costs, laser-assisted bioprinting achieves a resolution of 10–50 μm, handles high cell density (less than 10^8^ cells/mL), prints at 200–1600 mm/s, and maintains more than 95% cell viability post-print. It is capable of selecting single cells for transfer, which is a beneficial feature when precision is required to construct tissues [5]. In one example, Kerouredan et al. used a laser-assisted bioprinter to organize high density micropatterns of endothelial cells to form precise microvascular networks.

Stereolithography utilizes photopolymerizable liquid polymers that are crosslinked when exposed to UV, infrared, or visible light (a process called photocuring). It is a laser-assisted bioprinting system that works through exposure to the laser beam which photo-cures specified patterns and joins them in a layer-by-layer fashion. High resolution, low cost, and greater than 95% cell viability post-print are advantages of stereolithography while a noted limitation is the slow printing speed (15 mm/s) [24, 26, 27]. Stereolithography is ideal for printing personalized structures for disease modeling. For example, vessels with calcified plaque formation can be modeled prior to repair procedures to determine the most efficient pathways and techniques to relieve obstructions [28].

Scaffold-free bioprinting deposits spheroids on an array of needles to create tissues without the use of any ECM-based materials or scaffolds. Initially, the spheroids are cultured to optimum diameter and followed by single-layer extraction by the printer head. The spheroids are placed on the needle array in a layer-by-layer fashion and allowed to mature and fuse together. Finally, the resulting tissue is removed for further development in vivo, or for analysis [29]. Several aspects of scaffold-free bioprinting, including cost and cell viability, are variable and depend upon the technique used. For example, using an injectable cell suspension is cheaper than the more precise conventional cell sheet technology created by lowering the temperature of the culture dish. Furthermore, cell viability has been reported to be lower for cell suspensions and noted to be higher for cell sheets [30].

**Fig. 2:**
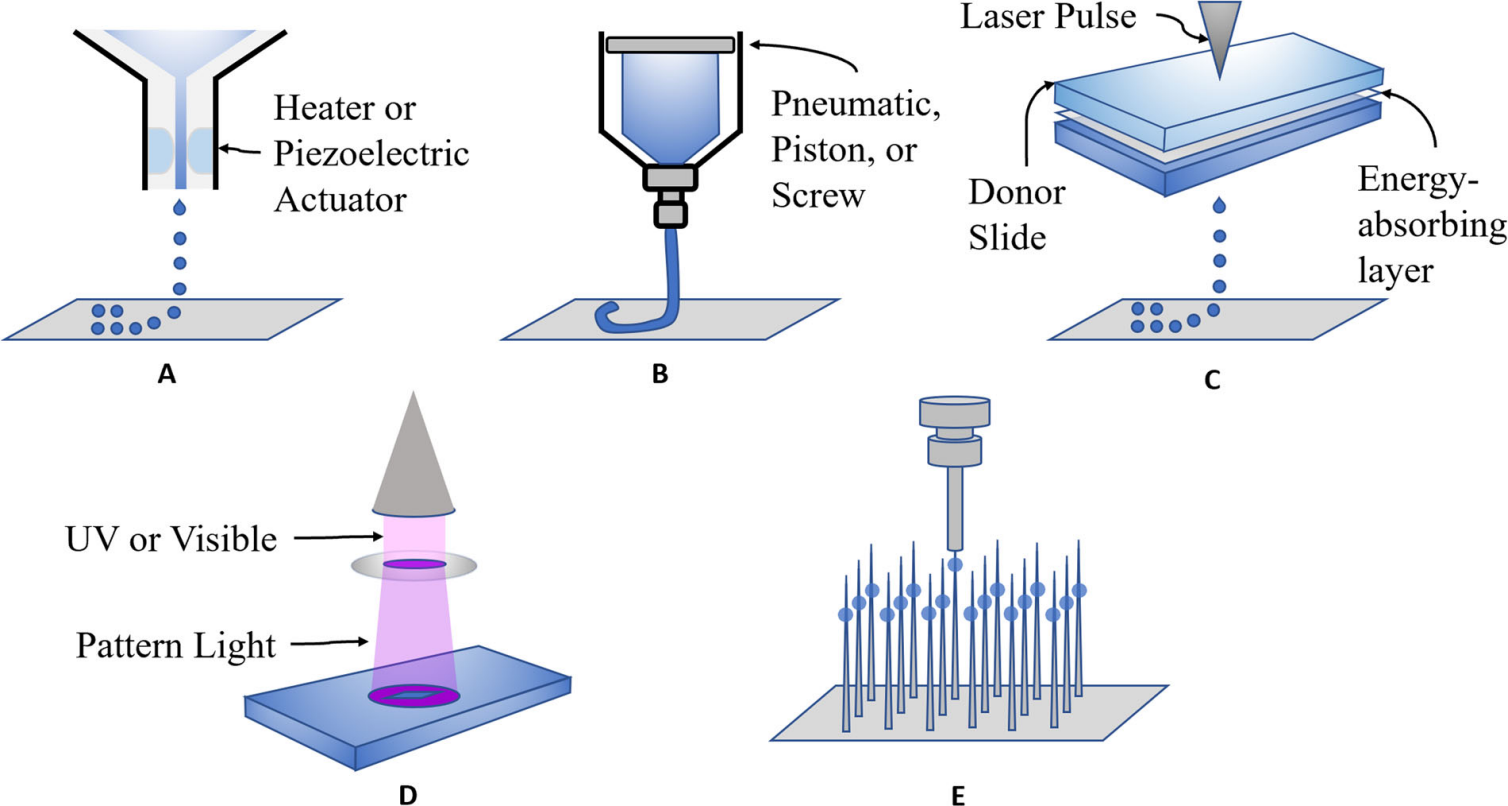
Bioprinting methods. **A** Inkjet printing. **B** Extrusion printing. **C** Laser-assisted printing. **D** Stereolithography. **E** Scaffold-free printing

Given the pros and cons of each technology, selecting the appropriate bioink and method is the determining factor in successfully creating functional cardiac tissue. An overview of various technologies used for 3D printing cardiac tissue is shown in Fig. 2 and the detailed specifications for each system are displayed in Table 1.

**Table 1:** Features of various 3D bioprinting methods

Method	Cell density	Cost	Resolution	Speed	Cell viability
Inkjet	<10^6^ cells/mL [5]	Low [5]	1–300 picoliter droplets, 50 um wide [5]	1–10,000 droplets per second [5]	~85% [23]
Extrusion	High (Cell spheroids) [24]	Moderate [24]	100 um [46]	Slow [24]	40–80% [24]
Laser-assisted	<10^8^ cells/mL [5]	High [5]	10–50 um [46]	Medium (200–1600 mm/s) [5]	>95% [23]
Stereolithography	25 × 10^6^ cells/mL [27]	Low [24]	5 um [26]	15 mm/s [26]	>95% [27]
Scaffold-free	Variable due to adjustable spheroid size [47]	Low (cell-suspension) to High (cell sheet) [30]	Low [30]	Medium to Long [30]	Low (cell-suspension) to High (cell sheet) [30]

## Bioinks for 3D printing

Biomaterials possess unique benefits to promote cell growth and proliferation, and performance can be improved through blending them with supplementary substances. A single material is usually not sufficient to foster tissue maturation, rather, a carefully selected amalgamation of ingredients is required for proper development [31, 32]. Alginate and gelatin are the two most common natural biomaterials extensively studied for cardiac tissue regeneration with 3D printing. Alginate is a naturally occurring polysaccharide commonly used to model cardiac tissue or for cardiac tissue engineering. The ability to be combined with diverse biomaterials improves the physical characteristics of alginate. Specifically, adding polyethylene glycol (PEG) fibrinogen to alginate allows the mixture to be stiffened through UV-light exposure which increases crosslinking and thus fine-tunes the mechanical properties of the material [31]. In addition, biocompatibility makes alginate an attractive material for tissue regeneration [32]. However, cell adherence to alginate is limited and is addressed by mosaicking cell adhesion moieties to create an environment that yields pre-tissue with desirable physical and chemical properties [31]. Maiullari et al. used hydrogel with alginate and PEG-fibrinogen to create a multicellular structure employing human umbilical vein endothelial cells (HUVECs) and induced pluripotent cell-derived cardiomyocytes (iPSC-CMs). The resulting tissue construct matured to vascular tissue in vivo.

Similarly, gelatin is a natural collagen-based extracellular matrix protein derived from mammals. Owing to the abundant cell attachment sites, gelatin based biomaterials promote cell proliferation, and mimic the native ECM environment enabling it to be an ideal material for fostering tissue development [33, 34]. Gelatin exists as liquid at 40 °C and gels below 40 °C which is beneficial for 3D bioprinting applications operating at normal body temperatures. Introduction of additional materials into gels and/or UV-induced cross-linking create a desired stiffness similar to alginate [35]. For example, Abudupataer et al. added methacrylic anhydride to a gelatin solution to synthesize gelatin methacryloyl (GelMA). Fibroblast cells were suspended in the gel to create cell laden GelMA which was bioprinted and cured using UV light for biomedical applications.

Decellularized extracellular matrix is derived from native tissues through the removal of cellular components from a variety of tissues/organs and has been used as a scaffold for cellular regeneration. Decellularized ECM is particularly useful in 3D bioprinting as it fosters cell survival and proliferation through inherent physical and mechanical characteristics. The native ECM structure of target tissue provides the signals required for proper function. Specifically, cells interact with the ECM through receptors while the ECM responds to the cellular behavior via mediators such as growth factors and enzymes [36]. Xu et al. developed blood vessels with intima, media, and adventitia layers using a 3D bioprinted scaffold with vascular channels by combining decellularized ECM and human aortic vascular smooth muscle cells. The decellularized ECM was perfused with pre-vascularized HUVECs to form the intima layer, and the adventitia layer was created using neonatal human dermal fibroblasts (HDF-n) to the outer portion of the 3D structure. This report shows that the natural characteristics of decellularized ECM facilitates the proper growth and function of seeded cells.

Nanoparticles are another component that can be added to hydrogels to enhance the biological properties of printed structures. For instance, Wei et al. included bioactive glass nanoparticles into an alginate-gelatin hydrogel to increase the stiffness of bioprinted mouse dermal fibroblasts. This augmented the strength of the printed material and improved cellular proliferation and adhesion. Considering that the mechanocompatibility of heart tissue, such approach is important to achieve adequate structural stability. Another useful characteristic of nanoparticles is the ability to manipulate post-injection into tissue. Lee et al. demonstrated this by developing nanovesicles derived from iron oxide nanoparticles that were injected into infarcted heart tissue. The study found that the retention of injected mesenchymal stem cells was enhanced by magnetic guidance. Furthermore, the inflammation phase of myocardial infarction was shifted into the reparative phase earlier; thus, increasing the potential for functional recovery. Further advancement in nanoparticles based bioprinting has been warranted in cardiac regeneration.

Diverse cell types are available for bioprinting applications including induced pluripotent stem cells (iPSCs), mesenchymal stem cells (MSCs), and embryonic stem cells (ESCs). Although ESCs develop into all three embryonic germ layers, researchers often use iPSCs because their use circumvents the ethical controversies regarding embryonic stem cells [37]. Induced pluripotent stem cells are created by reprogramming adult somatic cells such as fibroblasts to develop into any type of cell in the body and are often used in bioprinting. However, the particular iPSCs protocol used for reprogramming may affect development and hence a standard procedure promoting consistency is warranted [38]. MSCs are multipotent adult stem cells which facilitate the regeneration of multiple tissues [38, 39]. All these cell types can be combined with other biomaterials to promote the expression of desired phenotypes, as well as receive signals to differentiate into the lineages of interest [38]. For example, Kupfer et al. used gelatin methacrylate to formulate an ECM-based bioink to support proliferation and differentiation of hiPSCs into cardiomyocytes. Specifically, gelatin methacrylate was cross-linked with photoactivation, followed by mixing with ECM proteins and hiPSCs. The resulting material was used to bioprint a heart pump that exhibited superior electrochemical function and perfusion which sustained for 6 weeks. This method of using hiPSCs is amongst the most promising to date; however, further research is warranting.

Currently, efforts to generate functional cardiac tissues have utilized combinations of multiple biomaterials rather than a single type. Though efforts to date have yet to recapitulate all components of the adult native cardiac ECM in one construct, promising results have been achieved using different combinations of materials such as alginate, fibrinogen, gelatin, as well as decellularized ECM in conjunction with methods that include UV-induced crosslinking and methacrylation. A well-rounded list of properties and applications of biomaterials for cardiac tissue engineering is provided by Qasim et al. [40].

## 3D-printed cardiac constructs

The proper function of adult cardiac tissue is the result of a harmonious interplay between morphology, mechanics, electrophysiology, and gene expression. Although current studies have yet to recreate the desired characteristics of the native cardiac environment, many have engineered tissues with promising combinations and approaches (Table 2). For example, Das et al. combined decellularized left ventricle myocardial tissue with CMs from rats to create a bioink for the generatation of cardiac tissue constructs using extrusion-based printing. The created tissue was capable of producing a peak spontaneous force of ~13 μN, which is below the normal range of mature adult CMs (40–80 mN/mm^2^) [7]. However, the construct expressed type I/IV collagen and laminin, which are essential for the structural integrity of the cardiac tissue. Other studies that used similar materials have confirmed the expression of thesebiomarkers. For instance, Yu et al. used decellularized left ventricle tissue and hiPSC-CMs along with a hydrogel suspension to engineer cardiac tissue which upregulated expression of type I/IV collagen, laminin, fibronectin, glycosaminoglycans (GAGs), and myosin regulatory light chain 2 (a protein associated with movement of actin filaments). Moreover, the CMs were oriented in an organized fashion and capable of contracting; both features that are required to generate a directional force. These investigations suggest that hiPSC-CMs, decellularized ECM, and hydrogels strategically placed by an extrusion-based bioprinter can yield a tissue construct with native cardiac characteristics.

**Table 2:** Outcomes of cardiac tissue engineering studies

Parameters	Properties	Immature fetal CMs	Mature adult CMs	Belviso et al.	Lee et al.	Mannhardt et al.	Lemione et al.	Yu et al.	Das et al.	Noor et al.	Liu et al.	Ong et al.
Materials	–	–	–	Decellularized human skin (d-HuSk) as a scaffold for human cardiac progenitor cells (hCPCs)	Crosslinked gelatin hydrogels combined with hiPSC-CMs	Hydrogel combined with hiPSC-CMs	Hydrogel combined with hiPSC-CMs	Decellularized left ventricle heart tissue combined with hiPSC-CMs	Left ventricle myocardium decellularized ECM bioink combined with CMs	PSCs and ECM derived from fatty tissue. ECM was processed into a hydrogel	Human embryonic stem cell-derived CMs (hESC-CMs) printed into GelMA	hiPSC-CMs, fibroblasts, and endothelial cells combined to create spheroids
Printing method or technique	–	–	–	d-HuSk seeded with hCPCs	hiPSC-CM and hydrogel suspension placed in “casting mold”	hiPSC-CM and hydrogel suspension placed in “casting mold”	hiPSC-CM and hydrogel suspension placed in “casting mold”	hiPSC-CM and hydrogel suspension placed in “casting mold”	Extrusion-based printing	Extrusion-based printing	Light-based Micro-continuous Optical Printing	Biomaterial-free 3D bioprinter
Morphology	Cell shape	Circular [7]	Rod-shaped [7]	–	–	–	Rod-shaped	–	–	Elongated	–	–
	Cell organization	Disorganized [7]	Organized [7]	Organized	Organized	Organized	Organized	Organized	–	Organized	Depends on printing pattern: isotropic slabs or parallel lines	
Mechanical	Contractile force	0.08–4 mN/mm^2^ [7]	40–80 mN/mm^2^ [7]	–	0.3735 mN/mm^2^	~66 mN/mm^2^ ^b^	–	Contractility present but not measured.	Peak spontaneous force: ~13 μN (~34 mN/mm^2^) ^d^	Contractility present but not measured.	~0.09 mN/mm^2^ ^c^	Contractility present but not measured.
	Conduction velocity	~0.1–0.2 m/s [7]	~0.3–1 m/s [7]	–	–	–	–	Contractility present but not measured.	Contractility present but not measured.	>0.1 m/s	hESC-CMs printed in isotropic slabs yielded contraction without directional preference. Parallel lines contracted in the direction of patterning.	~0.04 m/s
Electrophysiology	Resting membrane potential	−20 to −60 mV [7]	−80 to −90 mV [7]	–	–	–	−73.5 ± 1.6 mV	–	–	–	–	–
	Upstroke velocity	10–50 V/s [7]	150–350 V/s [7]	–	–	–	219 ± 15 V/s	–	–	–	–	–
Expression	Collagen I	Type III » Type I [7]	2:1 (Type III: Type I)^a^ [7]	Present. Relative amounts not determined.	-	-	-	Present. Relative amounts not determined.	Present. Relative amounts not determined.	–	–	Collagen present. Type not indicated.
	Collagen III	Type III » Type I [7]	2:1 (Type III: Type I)^a^ [7]	Present. Relative amounts not determined.	–	–	–	–	–	–	–	Collagen present. Type not indicated.
	Collagen IV	largely absent [48]	Present in the basal membrane and along inner lining of T-tubules in ventricles. Abundant in ventricular ECM [39].	Present. Relative amounts not determined.	Present. Relative amounts not determined.	–	–	Present. Relative amounts not determined.	Present. Relative amounts not determined.	–	–	Collagen present. Type not indicated.
	Collagen VI	–	Fibers perpendicular to collagen type I & III [16]. Abundant in valvar ECM [39].	–	–	–	–	–	–	–	–	Collagen present. Type not indicated.
	Fibronectin	–	Present in the basal membrane and along inner lining of T-tubules in ventricles [7]	Present. Relative amounts not determined.	–	–	–	Present. Relative amounts not determined.	–	–	–	–
	Laminin	–	Present in the basal membrane and along inner lining of T-tubules in ventricles [7]	Present. Relative amounts not determined.	–	–	–	Present. Relative amounts not determined.	Present. Relative amounts not determined.	–	–	–
	Elastin	–	Fibers parallel to long axis of muscle fibers [7]	34.205 ± 2.529 μg/mg	Present. Elastin production increased with decreasing matrix stiffness.	–	–	–	–	–	–	–
	Glycosaminoglycans (GAGs)	–	5 types of GAGs detected: hyaluronic acid, heparan sulfate, dermatan sulfate, chondroitin-4-sulfate and chondroitin-6-sulfate [7].	76.89 ± 14.22 μg/mg	–	–	–	Reduced possibly due to detergent-based decellularization treatments.	–	–	–	–
	MLC2v & TBX18	TBX18 > MLC2v [7]	MLC2v > TBX18 [7]	–	–	MLC2v present. Relative amounts not determined.	–	MLC2v present. Relative amounts not determined.	–	–	–	–
	Connexin 43 (Cx43)	–	Present [49]	Present	Present	–	Present	–	–	–	–	Present

^a^Total collagen reduced compared to fetal

^b^Calculated as 0.152 mN/(π * 0.54 mm^2^), force/embryoid body surface area

^c^Calculated as 6.5 μN/(1.1 mm * 0.25 mm)^2^, force/pillar surface area

^d^Calculated as 13 μN/(π * (0.011 mm)^2^), force/average surface area of CM (Du) [50–68]

Belviso et al. utilized decellularized human skin (d-HuSk) as a scaffold for human cardiac progenitor cells (hCPCs) where the cell organization resembled native cardiac tissue with the upregulation of type I/III/IV collagen, fibronectin, laminin, elastin, GAGs, and connexin-43. The d-HuSk provided the site for cell attachment and retained the vessels necessary for circulation. Similarly, decellularized matrix components can be extracted, formed into a hydrogel, and combined with cells to provide structural support as well as aid in cell regulation. For example, Ng et al. cultured cardiac c-kit cells (a marker for stem cells in the adult heart) on decellularized MSC matrices [41]. Results showed that MSC-ECM provided resistance of cardiac c-kit cells to exogenous hydrogen peroxide – a component of CM progression to cell death. Protective effects were also observed when Wang et al. injected heart ventricles injured by myocardial infarction (MI) with neonatal mouse decellularized ECM. Following injection, measurements of cardiac contractile function showed significant improvement 6 weeks post-MI in comparison to controls. Furthermore, MI-induced fibrosis was significantly reduced contributing to increased heart function. These studies allude to the diverse uses and potential benefits of decellularized ECM for cardiac repair in vivo.

Tissue engineering methods often include the use of hydrogels due to their ability to retain 3D shapes as well as their similarities to the native ECM environment [42]. Noor et al. combined a hydrogel from fatty-tissue-derived ECM with pluripotent stem cells (PSCs) to print cardiac tissue using an extrusion-based bioprinter. The printed tissue construct exhibited contractility with a conduction velocity greater than 0.1 m/s which is in the range of immature fetal CMs (0.1–0.2 m/s). Despite the lower conduction velocity, the cells were organized and elongated suggesting a viable method of production for perfusable cardiac patches. Liu et al. used GelMA, in combination with human embryonic stem cells derived CMs (hESC-CMs) to produce cardiac tissue using a light-based micro-continuous optical printing method; specifically, GelMA with suspended hESC-CMs were printed in isotropic slabs or parallel lines, and then exposed to UV light to increase crosslinking. As the printed biomaterial matured, the cells formed an elongated shape and exhibited contractility within the range of immature fetal CMs. This printing method facilitates adjustable scaffold stiffness, contractility and cardiac tissue-like orientation supporting the creation of personalized cardiac constructs.

Although scaffolds are an important platform in creating 3D tissue constructs, novel strategies have brought to light the possibility of building scaffold-free cardiac constructs by placing cellular components in proximity to each other and allowing them to generate their own support. For instance, Ong et al. utilized a biomaterial-free 3D printing method in which hiPSC-CMs, fibroblasts, and endothelial cells were combined to create spheroids that were then loaded onto a needle array and allowed to fuse. Spontaneous beating of these cardiac patches was observed within 3 days of printing, and the spheroids were electrically connected with an average conduction velocity of 0.03 m/s. Low electrical transmission speed, weak mechanical characteristics and immature blood vessels were the major drawbacks of this method. Similarly, Arai et al. used different combinations of hiPSC-CMs, HUVECs, and normal human dermal fibroblasts (NHDFs) to generate scaffold-free tissue. Results showed optimal spheroid viability at a ratio of 50:25:25 mixture of hiPSC-CMs, HUVECs, and NHDFs, respectively. Each of these components played a crucial role in maintaining cell survival and functionality. For example, fibroblasts were shown to promote cell organization into spheroids, so they were added to the mixture. Although some disadvantages were observed, such as shrinkage of the construct after removal from the needle array, the final product was a functional scaffold free cardiac tubular construct. Another feature of scaffold-free tissue constructs is the ability to adjust spheroid cell density. Boyer et al. created scaffold-free microtissues using placental-derived MSCs where various spheroid cell concentrations were used to demonstrate the variability in characteristics of the tissue depending on cell density. The higher density spheroids tended to derive tissue with tighter cell-cell binding. Despite its limitations, scaffold-free bioprinting offers advantages such as high cell density and improved cell interactions–features that may resemble the native cardiac environment more closely than the methods involving scaffolds.

## Future directions

The major advantage of 3D cardiac tissue bioprinting is the potential to improve cardiac function without the need for donor implantation. Continued advancements in bioprinting methods can lead to improved performance and in vivo functionality of cardiac tissue constructs. Approaches such as injecting decellularized ECM-derived hydrogels into injured cardiac tissue have been effective in improving function after ischemia or MI [43]. Three-dimensional bioprinting technology has yet to make a significant impact on the field of clinical cardiology and clinical trials regarding the same are limited. Of note, in a recent clinical trial for VentriGel, a cardiac ECM hydrogel, 15 patients with moderate left ventricular dysfunction received up to 18 injections into the infarct and surrounding border areas. Patients that were treated post-MI showed improvements after one year in left ventricular remodeling, and all patients decreased in New York Heart Association (NYHA) functional class. Although further studies are needed to assess the efficacy of VentriGel, the results of this unprecedented study shed light on the potential benefits of 3D cardiac bioprinting [44]. Moreover, cardiac 3D bioprinting offers promising opportunities for micropatterning of the scaffold as well as scaffold-free systems to recreate the histological architecture of the native cardiac tissue. Perhaps the printing of customized tissue constructs according to a patient’s unique anatomy would enhance the beneficial effects of regenerative cardiology.

In addition, the introduction of 4-dimensional bioprinting has gained traction. This is a concept that utilizes 3D bioprinting to create an initial product that can be maneuvered into its final form over time. For example, moldable materials can be integrated into an initial 3D-bioprinted structure, and subsequently manipulated to create the desired shape, durability, and functionality for a specific purpose [45]. Though practical applications of 4D-bioprinting are currently under investigation, it has the potential to expand the tools available for fabricating intricate structures that closely mimic natural tissues. Bioprinting technology proceeds towards the development of clinically viable cardiac constructs for the management of MI and associated complications. However, the field of cardiac bioprinting is still in its infancy and further research is warranted to translate the encouraging laboratory findings into the clinical arena.
